# Minimally Invasive Sampling of Mediastinal Lesions

**DOI:** 10.3390/life14101291

**Published:** 2024-10-11

**Authors:** Alberto Fantin, Nadia Castaldo, Ernesto Crisafulli, Giulia Sartori, Alice Villa, Elide Felici, Stefano Kette, Filippo Patrucco, Erik H. F. M. van der Heijden, Paolo Vailati, Giuseppe Morana, Vincenzo Patruno

**Affiliations:** 1 Department of Pulmonology, S. Maria della Misericordia University Hospital, 33100 Udine, Italy; 2 Department of Medicine, Respiratory Medicine Unit, Azienda Ospedaliera Universitaria Integrata of Verona, University of Verona, 37134 Verona, Italy; 3 Pulmonology Unit, Department of Medical Surgical and Health Sciences, University Hospital of Cattinara, University of Trieste, 34149 Trieste, Italy; 4 Division of Respiratory Diseases, Department of Medicine, Maggiore della Carità University Hospital, 28100 Novara, Italy; 5 Department of Pulmonary Medicine, Radboud University Medical Center, 6525 GA Nijmegen, The Netherlands

**Keywords:** EBUS, EUS, cryobiopsy, TBNA, mediastinal, lymph node

## Abstract

This narrative review examines the existing literature on minimally invasive image-guided sampling techniques of mediastinal lesions gathered from international databases (Medline, PubMed, Scopus, and Google Scholar). Original studies, systematic reviews with meta-analyses, randomized controlled trials, and case reports published between January 2009 and November 2023 were included. Four authors independently conducted the search to minimize bias, removed duplicates, and selected and evaluated the studies. The review focuses on the recent advancements in mediastinal sampling techniques, including EBUS-TBNA, EUS-FNA and FNB, IFB, and nodal cryobiopsy. The review highlights the advantages of an integrated approach using these techniques for diagnosing and staging mediastinal diseases, which, when used competently, significantly increase diagnostic yield and accuracy.

## 1. Introduction

Mediastinal lesions represent a heterogeneous group of clinical entities that arise in the mediastinum, the central compartment of the thoracic cavity. These lesions encompass a broad spectrum of pathologies, including benign and malignant tumors, cysts, and inflammatory processes [1]. The mediastinum houses critical structures such as the heart, great vessels, trachea, esophagus, and lymph nodes, making diagnosing and managing mediastinal lesions diverse and particularly challenging [2,3,4,5]. This complexity is compounded by the anatomical intricacies of the region and the varied clinical presentations of mediastinal diseases, which can range from incidental findings in asymptomatic patients to severe symptoms caused by mass effect or invasion of adjacent structures [6,7,8,9].

The classification of mediastinal lesions is traditionally based on their anatomical location within the mediastinum, which is divided into the following three compartments: prevascular, visceral, and paravertebral [10]. Each compartment is associated with distinct types of lesions, influenced by the embryological origins of the tissues within these regions (see Table 1). For instance, thymomas, germ cell tumors, and lymphomas are commonly found in the prevascular compartment [11,12,13], whereas bronchogenic cysts, vascular lesions, esophageal diseases, and lymphadenopathy are more typical of the visceral one [14]. Lastly, neurogenic tumors, bone, and cartilage pathologies predominantly occupy the paravertebral compartment [15,16,17,18]. This compartmentalization aids in narrowing the differential diagnosis and guiding further diagnostic investigations.

Advancements in imaging modalities, particularly computed tomography (CT) and magnetic resonance imaging (MRI), have significantly enhanced the ability to detect and characterize mediastinal lesions [10,19,20,21]. These imaging techniques provide detailed anatomical information and can help with assessing the extent of the lesion, its relationship with surrounding structures, and potential invasion. Positron emission tomography (PET) combined with CT (PET/CT) has further improved the evaluation of mediastinal masses by providing metabolic information that can distinguish between benign and malignant lesions, as well as between the different types of malignancies [22].

Histopathological examination remains the gold standard for definitive diagnosis in most cases [1,23,24,25]. Various minimally invasive techniques have been developed to obtain tissue samples from mediastinal lesions, reducing the need for more invasive surgical procedures [26].

This article aims to provide a comprehensive review of the current techniques for minimally invasive sampling of mediastinal lesions (Table 2), encompassing their classification, diagnostic performances, and respective complications. By synthesizing recent advancements in the field, we seek to offer insights into the challenges and opportunities in diagnosing these complex conditions.

**Table 1:** Differential diagnosis of mediastinal lesions classified by the tissues of origin [11,12,27,28,29,30,31,32,33,34,35,36,37,38,39,40,41,42,43,44].

**Lymph Nodes**	**Pericardium**
Reactive hyperplasia Infection Metastasis Hodgkin lymphoma Non-Hodgkin lymphoma Sarcoidosis Lymphangioma	Cyst Pericardial recess Mesothelioma Sarcoma Fibroma Lymphoma Lipoma Teratoma Metastasis
**Esophagus**	**Heart**
Esophageal cyst Hiatus hernia Squamous cell carcinoma Adenocarcinoma Esophageal neuroendocrine tumors Leiomyoma Leiomyosarcoma Lymphoma Gastrointestinal stromal tumor Esophageal fibrovascular polyp Metastases	Myxoma Fibroma Fibroelastoma Lipoma Rabdomioma Hemangiomas Teratoma Amartoma Paraganglioma Sarcoma Metastasis
**Trachea, bronchi, and lung**	**Thymus**
Tracheal and bronchial cyst Papillomas Adenomas Amartoma Adenocarcinoma Squamous cell carcinoma Adenosquamous carcinoma Small cell lung cancer Large cell lung cancer Sarcomatoid carcinoma Lymphoma Rare neoplasms	Thymic cyst Thymoma Thymic carcinoma Thymic hyperplasia Squamous carcinoma Adenocarcinoma Adenosquamous carcinoma NUT carcinoma Salivary gland-like carcinomas Carcinoid Small cell carcinoma Large cell neuroendocrine carcinoma
**Pleura**	**Germ Cells**
Metastasis Mesothelioma Adenomatoid tumor Solitary fibrous tumor of the pleura Pleural angiosarcoma Pleural synovial sarcoma Desmoid-type fibromatosis of pleura Calcifying fibrous tumor of pleura Desmoplastic round cell tumor of pleura Lymphoma of the pleura	Seminoma Embryonal carcinoma Yolk sac tumor Choriocarcinoma Teratoma Mixed germ cell tumor Germ cell tumor with associated hematological malignancy
**Fibroblastic and myofibroblastic**	**Adipocitic**
Desmoid-type fibromatosis Solitary fibrous tumor Calcifying fibrous tumor Inflammatory myofibroblastic tumor Myxofibrosarcoma	Lipoma Thymolipoma/thymolipsarcoma Liposarcoma Lipoblastoma Mediastinal lipomatosis
**Vascular**	**Peripheral nerve sheath and nervous**
Aneurysm Anatomical variation Hemangioma Cavernous hemangioma Venous hemangioma Intramuscular hemangioma Arteriovenous hemangioma Lymphangioma Cystic lymphangioma Epithelioid hemangioendothelioma Angiosarcoma	Meningocele Extra-adrenal paraganglioma Granular cell tumor Schwannoma Malignant peripheral nerve sheath tumor Ganglioneuroma Ganglioneuroblastoma Neuroblastoma Paraganglioma Chemodectoma Pheochromocytoma
**Skeletal muscle**	**Bone and cartilage**
Rhabdomyosarcoma Embryonal rhabdomyosarcoma Spindle cell rhabdomyosarcoma Alveolar rhabdomyosarcoma Pleomorphic rhabdomyosarcoma	Osteophyte Osteoma Osteosarcoma Chondroma Chondrosarcoma Extramedullary hematopoiesis
**Thyroid lesions**	**Parathyroid lesions**
Thyroid nodule Goiter Thyroiditis Ectopic thyroid Papillary cancer Follicular cancer Medullary cancer Anaplastic cancer Metastasis	Adenoma Atypical parathyroid tumor Carcinoma Metastasis
**Others**	
Localized infection/Abscess Localized hemorrhage Mediastinitis Retroperitoneal recess Langerhans cell histiocytosis Erheim-Chester disease IgG4 disease Neuroenteric cyst Thoracic duct cyst	Mediastinal pancreatic pseudocyst Synovial sarcoma Spindle cell sarcoma Epithelioid cell sarcoma Biphasic synovial sarcoma Ewing sarcoma Round cell sarcoma Fluid extravasation from neighboring organs (e.g., enteral nutrition) Diaphragmatic hernia

**Table 2:** Comparison of sampling techniques. EBUS-IFB: EBUS-guided intranodal forceps biopsy; EBUS-TBNA: endobronchial ultrasound-guided transbronchial needle aspiration; EBUS-TMC: EBUS-transbronchial mediastinal cryobiopsy; EUS-B-Cryo: transesophageal cryobiopsy; EUS-FNA/FNB: endoscopic ultrasound fine needle aspiration and biopsy; EUS-B-FNA: trans-esophageal endobronchial ultrasound fine needle aspiration; PTNB: image-guided percutaneous transthoracic needle biopsy. (−): negative; (+/−): variable; (+) affirmative.

	EBUS-TBNA	EBUS-IFB	EBUS-TMC	EUS-FNA/FNB	EUS-B-FNA	EUS-B-Cryo	PTNB
Patient comfort	−	−	−	+	+	+	+
Sedation suggested	+	+	+	+	+	+	−
Need to access the central airways	+	+	+	−	−	−	−
Need to access the esophagus	−	−	−	+	+	+	−
Histological specimen	+/−	+	+	+/−	−	+	+

## 2. Methodology of the Review

This narrative review evaluates the existing literature by collecting primary English language bibliographic references from international scientific databases (Medline/Pubmed, Scopus, and Google Scholar). The search strategy aimed to include the most significant documents for the human species, dealing with the minimally invasive sampling of mediastinal lesions. The interval considered by the research was from January 2009 to November 2023. Systematic reviews, meta-analyses, randomized control trials (RCTs), original research papers, and case reports were included in our review (see Supplementary Materials for the complete search strategy and CONSORT diagram). The authors included other references that were considered significant.

To minimize bias, the authors independently searched, deduplicated the research results, screened the articles, and selected the studies to be included in this review using the Systematic Review Accelerator (https://sr-accelerator.com/). Of note, the list of included references is not necessarily an all-encompassing one, but it reflects the body of evidence believed to be appropriate for the purpose of this document—highlighting the latest progress made in the field.

## 3. Planning and Execution of the Procedure

A procedure aimed at sampling a mediastinal lesion entails a meticulously coordinated series of steps to ensure precision and safety [45,46,47]. Initially, the patient undergoes a comprehensive pre-procedural evaluation, including imaging studies such as magnetic resonance imaging, CT, or PET-CT to delineate the lesion’s size, location, and characteristics [1,48,49]. These imaging data are crucial for planning the route of approach, either through an endoscopic or percutaneous route. Laboratory tests such as cell blood count, coagulation tests, and kidney and liver function may be required. The procedure begins with the administration of local anesthesia and, eventually, sedation to facilitate patient comfort and cooperation [50,51,52]. 

If the procedural route is endoscopic, a flexible instrument is used to navigate through the airways or the digestive tract to the vicinity of the target lesion. Subsequently, imaging techniques confirm the precise location of the mediastinal lesion and detect the presence of critical anatomical structures that need to be avoided with the sampling tool [53,54,55]. Once the target is acquired and stabilized, the working channel of the endoscopic instrument accommodates the sampling tool of choice, which can be chosen based on the location of the lesion with respect to the endoscopic device, the radiologic morphology of the target (e.g., solid or necrotic), the diagnostic suspicion (e.g., benign or malignant pathology), and the tissues that are necessary to pass through with the tool before reaching the target lesion itself [56,57]. The sampling tool is then advanced through the tissue wall into the lesion with real-time imaging modalities such as ultrasound or fluoroscopy to ensure accurate sampling and to avoid injury to adjacent anatomical structures [58,59].

On the other hand, if the planned procedure is percutaneous, the process begins with positioning the patient in the most appropriate position (decubitus) for access to the lesion [60]. Subsequently, a real-time ultrasound or tomographic/fluoroscopic-guided identification of the lesion and surrounding anatomic structures is performed. The skin site corresponding to the safest access to the lesion can be marked for identification. The operator then performs a sterilization of the target area and the administration of local anesthesia. Under real-time ultrasound or fluoroscopic guidance, or, alternatively, with interval tomographic evaluation, a fine needle is inserted through the chest wall into the mediastinal lesion [59,61]. Once the needle reaches the lesion, tissue samples are acquired for analysis. Finally, the retrieval of one or more biopsy cores using a dedicated tool may be performed [62,63].

Multiple tissue samples may be obtained to increase diagnostic yield and minimize the need for repeated procedures [64,65]. The samples are then processed for cytological and histopathological examination, which is pivotal for diagnosing conditions such as malignancy, granulomatous diseases, or infectious processes [66,67,68]. Post-procedure, the patient is monitored for potential complications such as bleeding, pneumothorax, fever, desaturation, and persistent impaired cognitive status [69]. Depending on the local protocol, an ultrasound, fluoroscopic, radiographic, or tomographic evaluation may be performed after a specific time interval from the end of sampling to rule out the presence of iatrogenic pneumothorax or other complications [70,71,72,73,74]. 

## 4. Endoscopic Imaging Technologies for Sampling Guidance

### 4.1. Convex Probe Endobronchial Ultrasound (CP-EBUS)

**Figure 1:**
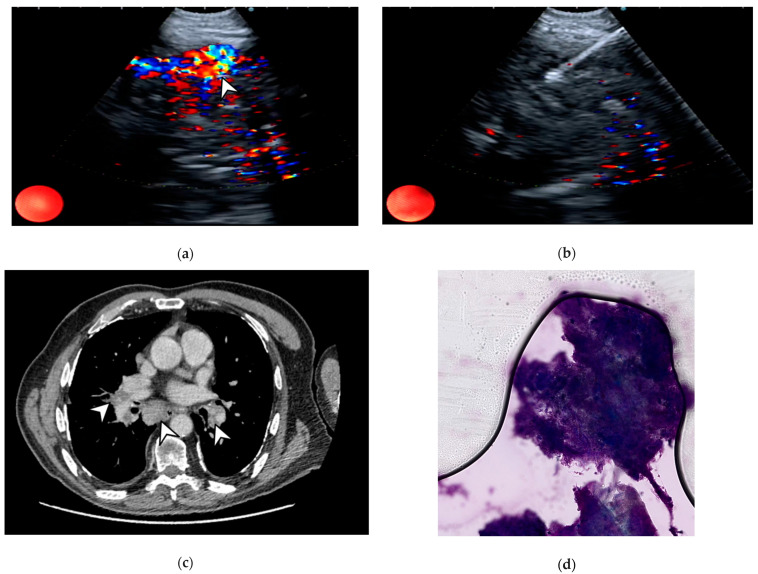
EBUS-TBNA of a subcarinal lymph node diagnostic for granuloma in a sarcoidosis case. (**a**) Echocolordoppler assessment via endobronchial ultrasound of the subcarinal lymph node demonstrates a large-caliber vessel’s presence (white arrowhead). (**b**) Sampling by EBUS-TBNA of the subcarinal lymph node. (**c**) Chest CT scan of the case under evaluation demonstrating mediastinal adenopathies at both lung hila and in the subcarinal station (white arrowheads). (**d**) Rapid on-site evaluation of the specimen demonstrating the presence of granulomas. The images are owned by the Department of Pulmonology, S. Maria della Misericordia University Hospital, Udine, Italy. Informed consent was obtained from the patients for the publication of the images in an anonymized format.

CP-EBUS is a medical procedure involving the insertion of an endoscope inside the lower airways (trachea and bronchi). It employs a flexible bronchoscope equipped with a convex ultrasound probe at its tip, which can generate high-resolution images of the surrounding tissues (see Figure 1). The probe emits ultrasound waves that penetrate the airway wall and create detailed cross-sectional images of the mediastinum and hilar structures [75]. A saline-filled inflated balloon may be used to cover the probe’s tip to expand the contact area between the device and the mucosa [45]. 

The investigated lesion’s ultrasound morphology may suggest the underlying etiology. In a retrospective analysis of 1061 images of lymph nodes in lung cancer patients, characteristics such as round shape, distinct margins, heterogeneous pattern, and the presence of necrosis emerged as independent predictors of malignant involvement [76]. Other criteria, such as a short axis larger than 1 cm, absence of central hilar structure, and high blood flow in a lymph node, are classified as high risk for malignancy. If less than three of these criteria are present, there are meager chances of the lymph node being malignant. The best single criterion to predict malignancy is heterogeneity [77].

The capacity of CP-EBUS to undertake sampling under direct ultrasound guidance is one of its main advantages. This feature increases the precision, diagnostic yield, and diagnostic accuracy of sampling techniques by guaranteeing accurate needle placement into target lesions [78]. 

### 4.2. Endoscopic Ultrasound (EUS)

EUS is a procedure that involves inserting a flexible endoscope with an ultrasound probe on its tip into the upper gastrointestinal tract [79]. EUS is a minimally invasive procedure used to evaluate the conditions affecting the gastrointestinal system and other adjacent organs and tissues [80,81]. 

Specific ultrasound features of target lesions, especially size and the presence of necrosis, are predictive of malignancy [82,83].

The advantages of EUS over CP-EBUS include a larger brightness mode window angle (EUS max 360 vs. EBUS max 60 degrees), a better ultrasonic image due to higher resolution, the ability to visualize small anatomical structures, better maneuverability of the endoscope in the various spatial planes, and close contact between the transducer and the target due to greater endoscopic suction with deflation of the esophageal lumen [84]. 

### 4.3. Trans-Esophageal Endobronchial Ultrasound (EUS-B)

EUS-B involves using a CP-EBUS bronchoscope inserted through the esophagus to sample a supra- or subdiaphragmatic lesion.

The technique has expanded the pulmonologist’s expertise, primarily in staging pulmonary neoplasms and diagnosing other mediastinal diseases [85,86,87,88].

### 4.4. Color Doppler

Doppler color flow imaging is a sophisticated imaging modality that improves the diagnostic power of traditional ultrasound. By visualizing blood flow within the region of interest, this approach can provide important insights into the vascular properties of both the target lesion and the surrounding tissues [89]. The obtained images display the direction and velocity of blood flow, allowing for the differentiation between vascular and non-vascular structures. The color Doppler feature is precious in assessing the vascularity of anatomical areas (e.g., chest wall), lymph nodes, and suspicious lesions, aiding in the differentiation between benign and malignant processes and in deciding the best route to the target and which area to sample within the target itself [90,91,92].

### 4.5. Strain Elastography

Elastography is an imaging technique found in modern ultrasound processors. This non-invasive method evaluates the stiffness and compressibility of tissue based on the knowledge that different tissues exhibit varying degrees of elasticity [93]. It may predict the differentiation between malignant and benign lymph nodes with sufficient sensibility, with malignant tissue typically exhibiting greater relative stiffness than normal ones [94]. 

By using a color scale resulting from compression deformation, elastography offers information on the stiffness of the tissues in the target area [95]. Typically, red regions indicate the softer portions of the sampled lesion, while blue parts are the stiffer ones. 

Thus, by avoiding the necrotic portions that may result in inadequate tissue sampling, elastography can make it easier to locate and sample the most representative portion of a solid lesion [96]. Alternatively, it may indicate the less stiff part of a hard or calcified fibrotic lymph node [97,98,99].

Nevertheless, it is crucial to emphasize that elastography does not replace the requirement of lymph node aspiration [100,101,102,103,104,105,106,107].

## 5. Percutaneous Imaging Technologies for Sampling Guidance

### 5.1. Transcutaneous Mediastinal Ultrasound (TMUS)

**Figure 2:**
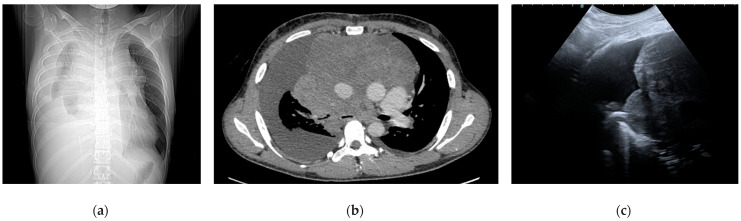
Primal mediastinal Hodgkin lymphoma. (**a**) Scout image demonstrating a right pleural effusion associated with mediastinal enlargement. (**b**) A contrast-enhanced tomographic image shows a large prevascular mediastinal mass with airway compression, heart dislodgement, and right pleural effusion. (**c**) Ultrasound appearance of the mass and the pleural effusion from a right parasternal view. The images are owned by the Department of Pulmonology, S. Maria della Misericordia University Hospital, Udine, Italy. Informed consent was obtained from the patients for the publication of the images in an anonymized format.

TMUS is a noteworthy development in non-invasive imaging methods for mediastinal structural assessment. With the use of a curvilinear or linear probe (1–7 vs. 5–20 MHz), TMUS offers real-time imaging of the chest and mediastinal anatomy [108,109,110,111] (see Figure 2). 

The benefits of TMUS over other methods described in this section are the lack of ionizing radiation, the affordability and portability of ultrasound devices, and the feasibility of doing a procedure at the patient’s bedside [112,113]. The disadvantages, especially when compared with computed tomography, are the condition of operator-dependence in image interpretation and the maximum tissue depth achievable with adequate resolution [114,115]. Contrast-enhanced ultrasound has been described in the setting of mediastinal sampling [116].

### 5.2. Fluoroscopy

Fluoroscopy is a real-time imaging technique using X-rays [117]. The fluoroscopic guide may be based on a basic fluoroscope or fluoroscopy derived from traditional computed tomography or cone beam computed tomography [118,119,120].

Depending on the inherent radio-opacity of the target lesion, the procedure may allow for proper placement of the sampling instrument within the lesion and real-time assessment of needle position relative to fluoroscopically identified lesion boundaries [59]. The significant advantage is that the radiologic evaluation occurs in real-time, resulting in the ability to quickly change the position of the sampling instrument [119]. The drawbacks are the radiological exposure of the patient [121] and operator [122], procedural limitations related to the radiological characteristics of the lesion [123], and the inability to identify, without the use of additional elements (e.g., contrast medium), structures adjacent to the lesion such as blood vessels [124].

### 5.3. Computed Tomography (CT)

CT is an advanced imaging modality that uses X-rays and computer processing to create detailed cross-sectional images of the body [125]. By obtaining intraprocedural high-resolution images, CT enables precise planning and significantly aids in preventing iatrogenic lung and vascular punctures. It guarantees high diagnostic yields (77–100%) in sampling mediastinal lesions [126,127,128]. 

The advantages include high spatial resolution, feasibility of three-dimensional reconstruction of the image, and the possibility of knowing the position of the sampling instrument in the three planes of space [63]. Disadvantages include the patient’s radiological exposure and the fact that guidance is not real-time, as clinical staff leaves the room during tomographic scans [129].

### 5.4. Magnetic Resonance Imaging (MRI)

MRI operates by using strong magnetic fields and radiofrequency pulses to align hydrogen nuclei in the tissues. This alignment creates detailed images of internal structures based on the different relaxation times of tissues when the magnetic field is altered [130]. Targeting subtle and inaccessible lesions may be possible using MRI’s multiplanar imaging capabilities, improved soft tissue contrast, and accurate portrayal of vessels [131].

The advantages include extremely high-quality resolution and a lack of ionizing radiation [132]. 

The main disadvantages are the high acquisition costs, longer procedural times, and the need to use dedicated sampling tools to prevent paramagnetic artifacts [133].

## 6. Sampling Techniques

### 6.1. Transbronchial Needle Aspiration (TBNA)

TBNA is a diagnostic technique where a dedicated needle is inserted through the operating channel of a bronchoscope and, by passing through the bronchial wall, allows for the acquisition of both cytological material and tissue cores [78]. The bronchoscope may be inserted through either the nasal or oral route [134]. Both endobronchial ultrasound-guided transbronchial needle aspiration (EBUS-TBNA) and conventional transbronchial needle aspiration (c-TBNA) are valuable diagnostic tools that may be used accordingly to local availability [135,136].

The accessible mediastinal lymph node stations through this route include paratracheal (2R, 2L, 4R, 4L), subcarinal (7), hilar (10R, 10L, 11R, 11L), lobar (12R, 12L), and segmental and subsegmental stations (13R, 13L, 14R, 14L) [137]. All mediastinal lesions with an edge in contact with the central airways may be sampled [46,54,138,139].

EBUS-guided TBNA performs optimally in malignant diseases involving the mediastinum [140]. It has a sensitivity of 88.0%, with 100% accuracy in establishing a diagnosis of lung cancer, exceeding the observed diagnostic results of cervical mediastinoscopy in randomized trials [141] while guaranteeing a lower rate of complications [142,143]. Confirmatory mediastinoscopy after a negative systematic endosonographic evaluation slightly reduces the rate of unforeseen N2/3 disease but with a high needed to treat number of patients (N = 23.8) [144], and it is avoidable in almost all patients [145].

Benign, hematological, and rare diseases of the mediastinum may represent a weak spot of TBNA as the quality of the specimen and the absence of an actual histological sample may reduce the diagnostic yield for these entities [146,147]. Currently, the reported diagnostic yield for sarcoidosis in actual clinical practice is 79%, with sensitivity reaching 84% in high procedural volume centers [148]. For tuberculous lymphadenitis, the diagnostic yield of EBUS-TBNA is 80% [149,150].

Since the advent of EBUS-TBNA, the optimal needle size has been under discussion and remains a topic of ongoing interest. The primary objective in considering different needle sizes is the requirement to obtain a sufficient amount of high-quality tissue without provoking adverse events. Different needle sizes are available; the most frequently used in clinical practice and studied in the literature are the 21G, 22G, and 19G. The overall EBUS-TBNA sensitivity is, respectively, 93% for the 19G needle, 87.6% for the 21G needle, and 85% for the 22G needle [151]. The overall sensitivity of EBUS-TBNA for diagnosing NSCLC is 92.9% for the 19G needle, 89.4% for the 21G needle, and 82.1% for the 22G needle [151]. The general conclusion currently accepted by the scientific community is that the 19G, 21G, and 22G needles have comparably excellent diagnostic sensitivity [151,152,153,154,155,156]. 

Kassirian et al. evaluated, in a systematic review and meta-analysis, a pool of 4242 patients to esteem the diagnostic yield of different needle sizes aimed at diagnosing sarcoidosis. The 19G group demonstrated a significantly higher (93.73%) sensitivity when compared to the 21G (84.61%) and 22G groups (84.07%) [157].

New 22G biopsy needles with a side-cutting window have been evaluated; unfortunately, they do not demonstrate a sensibility benefit in patients with benign mediastinal diseases such as sarcoidosis [158]. Additionally, a new crown-cut needle technology (Franseen needle) has been tested with encouraging results, especially in benign mediastinal diseases, despite equivalence in diagnostic accuracy, guaranteeing a better sample quality than conventional needles [159].

A 2012 RCT showed no additional benefit in terms of sample quality, diagnostic yield, or accuracy derived from applying needle suction while performing samples via EBUS-TBNA [160]. The results were later confirmed in subsequent studies for both the rapid on-site and final pathological evaluations [161,162]. Notwithstanding, a recent RCT demonstrated the possibility of a benefit on the diagnostic yield of the cellblocks and not the smears while applying suction [163]. 

Additional research shows how omitting stylet use during EBUS-TBNA does not impact diagnostic outcomes and decreases procedural complexity [164,165]. Lastly, the number of needle passes does not affect diagnostic yield for benign conditions after ten revolutions [166], while increasing needle passes improves the yield for next-generation sequencing for lung cancer [167].

EBUS-TBNA is a safe procedure with an overall complication rate approaching 1.4% [78,168]. Bleeding is the most frequently described complication but requires dedicated intervention in only 0.2% of procedures [169,170]. Isolated cases of mortality associated with EBUS-TBNA and delayed and fatal bleeding have been described [171]. Pneumothorax (0.53%) may arise as a complication, but it does not always necessitate the placement of a chest drain [169,170,172]. Mediastinal infections (0.10%) and pneumonia (0.22%) have been described [170,173,174,175,176]. Acute respiratory failure (0.3%) and the exacerbation of existing diseases are rare [169,170]. The incidence of complications increases in patients older than 70 years [169]. Needle breakage is rare but has been described [177,178,179,180].

### 6.2. EBUS-Guided Intranodal Forceps Biopsy (EBUS-IFB)

EBUS-IFB is a complimentary procedure, usually performed following EBUS-TBNA, and involves using biopsy forceps within the operating channel of the EBUS bronchoscope (see Video S1) [181]. Through the breach previously generated by the needle itself, the forceps are advanced inside the target lesion to take a histologic specimen from it [182]. In the past, large gauge needles containing biopsy forceps within their structural architecture were investigated; however, they have been poorly integrated into clinical practice since then. In 2012, a pilot study by Herth et al. proved that using 1.5 mm mini-forceps to obtain tissue for the diagnosis of enlarged mediastinal lymphadenopathy was a safe and feasible technique, providing a diagnosis yield of 86% [183]. 

The recent literature has described the use of different-sized forceps (0.96–1.9 mm) that were also applied in sampling peripheral lung lesions [184]. There was initial evidence that larger forceps provided better sample quality and size, as well as greater diagnostic yield [185].

A systematic review and meta-analysis by Agrawal et al. considering 443 patients showed an overall diagnostic yield in the diagnosis of intrathoracic adenopathy of 92% for EBUS-TBNA combined with EBUS-IFB and only 67% for EBUS-TBNA alone. A subgroup analysis of the same cohort showed an increased diagnostic yield for lymphoma (86% vs. 30%) and sarcoidosis (93% vs. 58) using EBUS-TBNA combined with EBUS-IFB [186]. To enhance the histopathological evaluation results, the integration of EBUS-IFB as a supplementary approach may be considered [187]. 

The overall complication rate is 1.5%, including pneumomediastinum (1%), hemorrhage (0.8%), and respiratory failure (0.6%) [181,186]. A single report on forceps malfunction has been published [188]. 

### 6.3. EBUS-Transbronchial Mediastinal Cryobiopsy (EBUS-TMC)

EBUS-TMC is a novel procedure that uses various caliber freezing probes aimed at acquiring histological samples from mediastinal lesions [189]. This technique allows larger and more intact tissue volumes to be acquired, minimizing crush artifacts [189].

EBUS-TMC can be used either as a stand-alone technique or associated with EBUS-TBNA. In the case of a stand-alone sampling technique, the breach through the airway is generated with a high-frequency knife or a laser [190,191,192]; conversely, in the case of employment complementary to EBUS-TBNA, the freezing probe is inserted into the target lesion through the needle breach [193,194,195,196]. The 1.1 mm [193,194,195,196] probe and the 1.7 mm [194,195] probe are the most widely used, and the activation time of the workstation varies from 3 to 7 seconds for each sample [89]. The extremely low temperatures generated induce the specimen to adhere to the probe, allowing for its retrieval and en-bloc removal with the bronchoscope. 

This technique enhances diagnostic accuracy in various mediastinal diseases, especially in non-malignant conditions and rare clinical entities [189,197]. In a systematic review enrolling 555 patients, EBUS-TMC demonstrated a diagnostic advantage compared to EBUS-TBNA in lymphomas, non-pulmonary carcinomas, and benign diseases. For lymphoma, EBUS-TMC was diagnostic in 87% of cases compared to only 12% for EBUS-TBNA; it also allowed for the characterization of every lymphoma subtype [198]. In the same systematic review, the authors described the improvement in sample adequacy (97% for EBUS-TMC vs. 79% for EBUS-TBNA) for obtaining a complete genetic and immunohistochemical typization in the setting of lung cancer [196,198]. 

An RCT by Fan et al. compared a group of patients undergoing combined EBUS-TBNA and EBUS-TMC with another group undergoing EBUS-TBNA alone [192]. They enrolled 271 patients with two coprimary endpoints—procedure-related complications and diagnostic yield. EBUS-TMC demonstrated a good safety profile and a notable increase in the diagnostic yield (93% in the combined group vs. 81% in the control group) for mediastinal lesions [192]. The combined approach improved the adequacy of tissue samples for molecular and immunological analyses of lung cancer. The incidence of complications did not differ between the groups [192].

The most frequent complication is bleeding, described in up to 85% of cases [89,199], currently justifying the use of a secured airway during the procedure.

### 6.4. Endoscopic Ultrasound Fine Needle Aspiration and Biopsy (EUS-FNA and FNB)

EUS-FNA and EUS-FNB are pivotal techniques for sampling mediastinal lesions [200]. EUS-FNA uses a fine needle to aspirate cells for cytological examination, while EUS-FNB employs a core needle to obtain tissue samples for histological analysis [201].

Compared with EBUS-TBNA, EUS-FNA/FNB has a greater extent of feasible mediastinal sampling sites. For example, lymph node stations 8 and 9 can also be reached, making the systematic staging of the mediastinum more comprehensive during the staging of chest oncological diseases [202,203]. 

EUS-FNA is widely used worldwide by gastroenterology specialists and occasionally by pulmonologists or surgeons due to its high diagnostic yield [204,205,206,207,208]. However, its reliance on cytological assessment can be limiting in cases where architectural tissue information is crucial. Conversely, EUS-FNB provides core tissue samples that preserve histological architecture, facilitating comprehensive pathological assessment [209]. Comparative studies for abdominal lesions indicate that EUS-FNB generally offers a superior diagnostic yield and accuracy compared to EUS-FNA [210]. Additionally, the diagnostic accuracy of EUS-FNB is slightly higher, often exceeding 90% [211], due to its ability to provide more adequate tissue samples. Renelus et al.’s meta-analysis revealed that EUS-FNB outperformed EUS-FNA for diagnostic yield (87% vs. 81%) [212]. Similarly, another systematic review and meta-analysis by Van Riet et al. discovered that the EUS-FNB outperformed EUS-FNA accuracy in 14 RCTs involving malignant and non-malignant lesions (87% vs. 80%) [213]. Lastly, a network meta-analysis by Han et al. that included both malignant and non-malignant lesions likewise showed the superiority of EUS-FNB over EUS-FNA [214]. For diagnostic outcomes on mediastinal lesions, there are undoubtedly less data available than for sampling abdominal ones; however, the literature describes, in malignant mediastinal disease, a diagnostic accuracy equal to 87.5% or more for EUS-FNA [204,215,216,217] and a diagnostic accuracy of 97.0% for EUS-FNB [218], with high variability between the different cohorts [219].

Various needles are utilized in EUS procedures. For EUS-FNA, standard 22G and 25G needles are commonly used, with the latter often preferred for increased flexibility and reduced risk of blood contamination [220,221]. Larger 19G needles are available but seem comparable to 22G in terms of diagnostic results for the mediastinal region [219,222,223]. EUS-FNB employs specialized needles, such as Franseen, reverse-bevel, Menghini-tip, and fork-tip designs, which have been shown to improve sampling quality and diagnostic yield [224]. 

Applying suction to the needle versus a stylet slow-pull technique does not alter the diagnostic results or the bloodiness of samples [225]. Avoiding suction may provide better accuracy while using smaller needles, such as the 25G [226].

EUS-FNA and FNB are both safe procedures with a low cumulative complication rate (2.5%) and mortality rate (0.02%) [227,228], even for mediastinal sampling [229]. The main complications described for EUS-FNA of the mediastinal lymph nodes and cystic lesions are infections [228,230,231]; thus, caution is advised on the indication to the sample liquid or necrotic lesions of the mediastinum [232]. 

### 6.5. Trans-Esophageal Endobronchial Ultrasound Fine Needle Aspiration (EUS-B-FNA)

In this technique, the EBUS bronchoscope is introduced into the esophageal route until it reaches the stomach, enabling the visualization of mediastinal and thoracic para-esophageal structures. Subsequently, a fine needle is used to sample the identified lesion.

EBUS-TBNA and EUS-B-FNA can be considered complementary techniques that can be performed consequentially during the same procedure using a bronchoscope. In recent years, a flexible bronchoscope has been proposed in both the airway and digestive tract evaluation phases, especially as a lung cancer staging procedure [186]. The use of the two techniques makes it possible to reach mediastinal locations that cannot be hit with either one alone, such as mediastinal and retroperitoneal lymph nodes [87,233], lung and pleural masses [234,235,236,237], pericardial fluid [238], ascites [239] and lesions in the liver [240], pancreas [241] or left adrenal gland [46,242]. 

An RCT by Madan et al. recruited 100 patients to compare the diagnostic yield and patient comfort between an EBUS-TBNA group and an EUS-B-FNA one in sampling mediastinal lymphadenopathy [88]. The diagnostic yield and adequacy of the aspirates were comparable across the two groups. However, with the EUS-B-FNA technique, the operator evaluated the intraprocedural patient’s comfort substantially higher with significantly less cough [88]. With EUS-B-FNA, the procedure took less time than EBUS-TBNA alone (16.4 versus 18.1 min). The authors concluded that for undiagnosed mediastinal lymphadenopathy located predominantly at the subcarinal or lower left paratracheal stations, EUS-B-FNA, compared with EBUS-TBNA, provides greater patient comfort with a similar diagnostic yield [88].

EUS-B-FNA can be considered an ideal approach in patients with severely compromised lower airways or respiratory failure requiring a mediastinal sampling procedure as the bronchoscope does not obstruct the airway or risk worsening the patient’s clinical condition [243]. 

To this day, the evidence still suggests that combining EBUS-TBNA with EUS-FNA is associated with better diagnostic accuracy than combining EBUS-TBNA with EUS-B-FNA [244]. This can be explained by the recent introduction of EUS-B-FNA into clinical practice and the need for practitioners to refine the technique and maintain sufficient operative volumes to maintain technical skills. Additionally, significant technical differences remained between the two procedures and utilized devices, primarily the size and maneuverability of the instrument, which is undoubtedly greater for a gastroscope than a bronchoscope, as well as the ability to insufflate air within the lumen of the digestive tract to locate anatomical landmarks, which is not feasible with a bronchoscope, implying that the proceduralist must orient himself just with the endoluminal ultrasound guidance.

EUS-B-FNA alone or in combination with EBUS-TBNA can also be performed in the pediatric population, as it has been proven safe [245]. The types of adverse events are similar to those of EUS-FNA.

### 6.6. Transesophageal Cryobiopsy (EUS-B-Cryo)

EUS-B-Cryo is a recently introduced procedure involving a freezing probe, used through a bronchoscope under EBUS guidance through the esophageal tissue layers [246]. Candidate patients for this procedure described in the literature generally have respiratory failure that does not allow safe passage of the bronchoscope through the airway without high risk to the patient [246].

The route for the freezing probe is usually created by previous FNA sampling of the target lesion [246,247]; however, the use of a high-frequency knife has been described [248].

The evidence favoring this procedure is mainly based on case-reports, and complications still have not been well described and ascertained because the follow-up periods of patients have been very short.

### 6.7. Image-Guided Percutaneous Transthoracic Needle Biopsy (PTNB)

**Figure 3:**
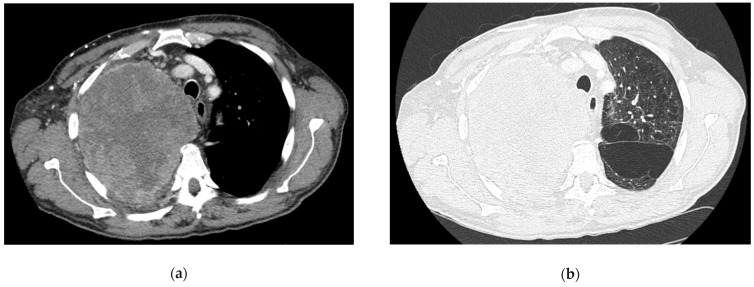

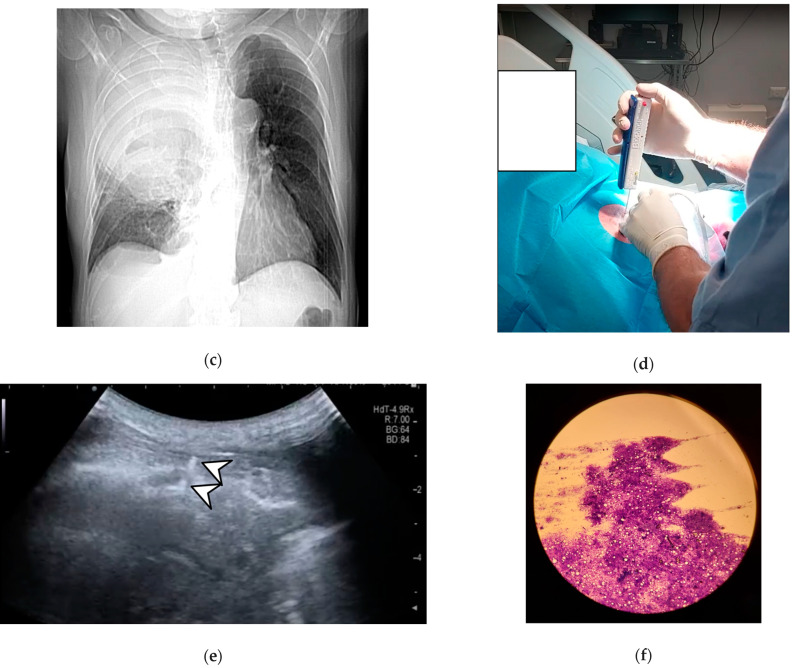
Percutaneous sampling with ultrasound guidance. (**a**) A contrast-enhanced tomographic image shows a large mass with mediastinal infiltration. (**b**) Tomographic image showing the mass, emphysematous changes in contralateral lung parenchyma and airway deviation. (**c**) Scout image demonstrates the extent of the mass. (**d**) Ergonomics of percutaneous sampling with the patient in supine decubitus, one operator’s hand holding the biopsy needle and the other hand holding the ultrasound probe covered with a sterile sheath. (**e**) Ultrasound appearance of the needle inside the lesion (white arrowheads). (**f**) Rapid on-site evaluation of the specimen demonstrates a sample with a high proportion of necrotic tissue. The images are owned by the Department of Pulmonology, S. Maria della Misericordia University Hospital, Udine, Italy. Informed consent was obtained from the patients for the publication of the images in an anonymized format.

Percutaneous transthoracic needle biopsy (PTNB) involves the insertion of a needle through the chest wall to reach and acquire the target mediastinal tissue (see Figure 3). 

The following two types of PTNB exist: fine-needle aspiration (FNA) and core needle biopsy (CNB). FNA uses a thin needle to acquire a cytological specimen from the lesion, whereas CNB uses a larger, hollow needle to obtain larger core tissue samples for histology [249,250]. The indication to use only one or both sampling modalities depends on the suspected nature of the lesion and the foreseeable need for a histological specimen [251]. CNB has a higher sample adequacy (89.6% vs. 75.5%) and diagnostic yield (81.3% vs. 53.1%) when compared with FNA [251].

The access route for small- to medium-caliber lesions is preferably extra pleural with a parasternal route and, in rare cases, paravertebral, trans-sternal, or suprasternal [252]. Another option is the transpulmonary technique, which entails the needle passing into the lung and visceral pleura with an increased risk of complications [253].

Of the various imaging modalities for sampling, TMUS, when feasible, provides the best sampling success over CT for both pulmonary and pleural lesions [254] with reduced procedural times and complication rates [129]; there is initial evidence with similar conclusions regarding sampling of mediastinal lesions as well [112]. 

The complication rate for mediastinal PTNB is 4.5–11% [119,255]. Described complications include pneumothorax (2%), bleeding (0.3%), and pleural and chest wall tumoral seeding [112,256]. 

## 7. Rapid On-Site Evaluation (ROSE)

ROSE is an emergent diagnostic tool in cytopathology that improves diagnostic procedures by analyzing intraprocedural cytological samples and imprinted bioptical ones [257,258].

ROSE involves the immediate microscopic examination of the acquired samples under a microscope or a slide scanner at the time and site of the procedure by a cytopathologist or other qualified operator [259,260]. This prompt assessment guarantees the sufficiency of the gathered specimen, thus diminishing the necessity for additional procedures, mitigating procedural and anesthesia time, and reducing the complication rate [261,262]. Additionally, ROSE makes quick preliminary diagnosis feasible, which could shorten the time from starting the diagnostic pathway to establishing dedicated therapy [263,264]. 

Finding ourselves at a time in history when mediastinal sampling modalities are plenty and have a non-negligible cost, ROSE can be employed to decide whether to conclude the procedure using a single low-cost sampling device (e.g., needle) versus proceeding to sample a lesion with a multimodal technique that employs several tools and increases procedural costs [265,266].

## 8. Conclusions

The correct and best route to sample a mediastinal lesion will likely be specific to the individual patient and the operating facility where the procedure is performed. The route by which sampling is conducted and the tools to be used must be identified prior to the procedure and possibly adjusted during it based on the imaging, clinical conditions, instrumentation available, and ROSE feedback [267,268]. 

The limitation of this review is the heterogeneity of the diagnostic endpoints used in the various bibliographic sources consulted.

The topic of mediastinal lesion sampling is a lively field of research, which is sure to see rapid evolution over time in the coming years. The available technology is already being further improved through the miniaturization of devices, the development of robotic endoscopy, and more sophisticated sampling techniques. The clinical relevance of mastering these procedures is to have a definite impact on the diagnostic and staging pathway of patients with malignant and benign mediastinal diseases.

## Supplementary Materials

The following supporting information can be downloaded at: https://www.mdpi.com/article/10.3390/life14101291/s1, Video S1: intranodal forceps biopsy sampling. Table S1. Search strategy used for the databases. Figure S1. CONSORT flow diagram of the review process.
