# Supplementary material for: Minimally Invasive Sampling of Mediastinal Lesions
Source: Life (Basel). 2024 Oct 11;14(10):1291. doi: 10.3390/life14101291 (PMC11509195; doi:10.3390/life14101291)
Supplement: Supplementary file 1 [file life-14-01291-s001.zip › Supplementary file - Search strategy.pdf]

Table S1. Search strategy used for the databases.

(Accessed by using University of Udine - VPN). Search performed on: Gen, 02, 2024

Databases	Group	Search Syntax	Records
**PubMed/ Medline**	1	(interventional pulmonology[MeSH Terms]) OR (lung cancer[MeSH Terms]) OR (bronchoscopy[MeSH Terms]) OR (endobronchial ultrasound[MeSH Terms]) OR (endoscopic ultrasound[MeSH Terms]) OR (EBUS[MeSH Terms]) OR (EUS[MeSH Terms]) OR (EUS-B[MeSH Terms]) OR (TBNA[MeSH Terms]) OR (TBFB[MeSH Terms]) OR (intranodal forceps biopsy[MeSH Terms]) OR (mediastinal cryobiopsy[MeSH Terms]) OR (lymph nodal cryobiopsy[MeSH Terms]) OR (esophageal[MeSH Terms]) AND (Mediastinal[Title/Abstract] OR Mediastinum[Title/Abstract])	**2.026**
**Scopus**	1	TITLE-ABS-KEY (“mediastinal OR mediastinum”)	**33.448**
	2	TITLE-ABS-KEY (“"interventional pulmonology" OR "lung cancer" OR bronchoscopy OR mediastinal OR "endobronchial ultrasound" OR endoscopic AND ultrasound OR ebus OR eus OR eus-b OR tbna OR tbb OR "intranodal forceps biopsy" OR "mediastinal cryobiopsy" OR "lymph nodal cryobiopsy" OR esophageal) in All fields	**124.349**
	**#1 AND #2**		**10.397**
**Google scholar**	1	“interventional pulmonology" OR "lung cancer" OR bronchoscopy OR "endobronchial ultrasound" OR endoscopic AND ultrasound OR ebus OR eus OR eus-b OR tbna OR tbb OR "intranodal forceps biopsy" OR "mediastinal cryobiopsy" OR "lymph nodal cryobiopsy" OR esophageal AND mediastinum OR mediastinal	**25.200**
		**Total evaluated**	**37.623**

Selection criteria:

- Time range: January 2009 – November 2023;
- Language: English;
- Species: Humans;
- Subject area: Medicine, Health professions;
- Type of study: systematic reviews, meta-analyses, randomized control trials, original research papers and case reports.

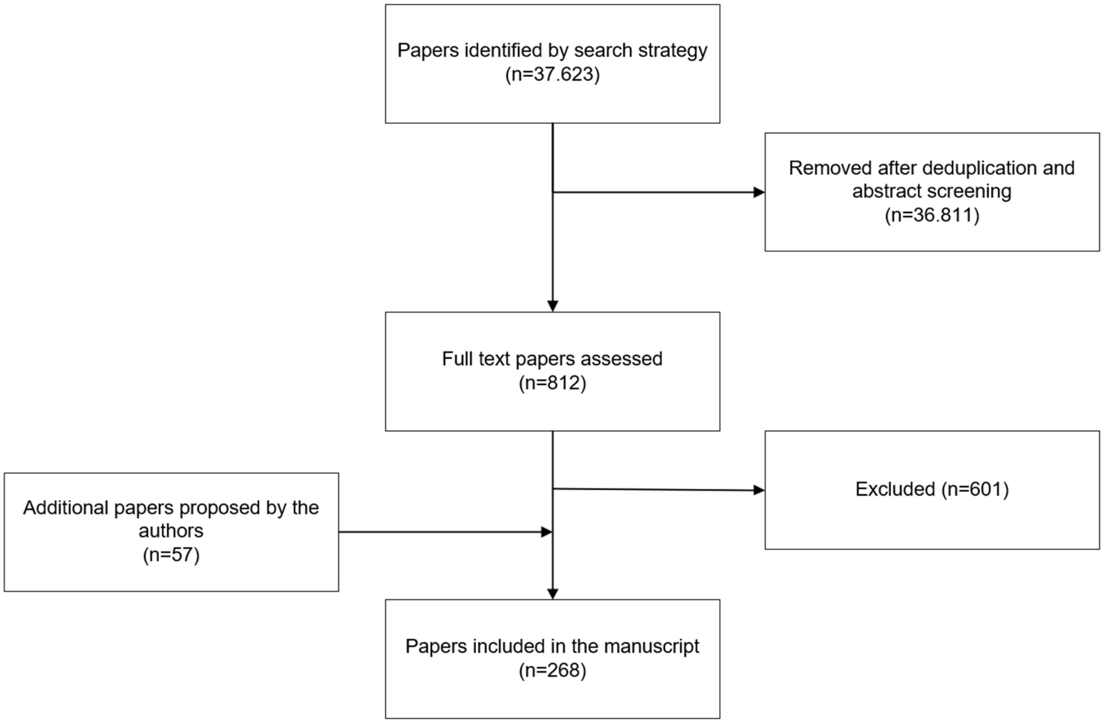

Figure S1. CONSORT flow diagram of the review process.
